# Analytical Formulation of the Electric Field Induced by Electrode Arrays: Towards Automated Dielectrophoretic Cell Sorting

**DOI:** 10.3390/mi8080253

**Published:** 2017-08-17

**Authors:** Vladimir Gauthier, Aude Bolopion, Michaël Gauthier

**Affiliations:** FEMTO-ST Institute, AS2M Department, Univ Bourgogne Franche-Comté, CNRS, 24 rue Alain Savary, 25000 Besancon, France; aude.bolopion@femto-st.fr (A.B.); michael.gauthier@femto-st.fr (M.G.)

**Keywords:** dielectrophoresis, micromanipulation, fourier series, electrode array, cell sorting

## Abstract

Dielectrophoresis is defined as the motion of an electrically polarisable particle in a non-uniform electric field. Current dielectrophoretic devices enabling sorting of cells are mostly controlled in open-loop applying a predefined voltage on micro-electrodes. Closed-loop control of these devices would enable to get advanced functionalities and also more robust behavior. Currently, the numerical models of dielectrophoretic force are too complex to be used in real-time closed-loop control. The aim of this paper is to propose a new type of models usable in this framework. We propose an analytical model of the electric field based on Fourier series to compute the dielectrophoretic force produced by parallel electrode arrays. Indeed, this method provides an analytical expression of the electric potential which decouples the geometrical factors (parameter of our system), the voltages applied on electrodes (input of our system), and the position of the cells (output of our system). Considering the Newton laws on each cell, it enables to generate easily a dynamic model of the cell positions (output) function of the voltages on electrodes (input). This dynamic model of our system is required to design the future closed-loop control law. The predicted dielectrophoretic forces are compared to a numerical simulation based on finite element model using COMSOL software. The model presented in this paper enables to compute the dielectrophoretic force applied to a cell by an electrode array in a few tenths of milliseconds. This model could be consequently used in future works for closed-loop control of dielectrophoretic devices.

## 1. Introduction

Cell analysis and cell sorting are of great interest for numerous biological applications [1,2,3]. Dielectrophoresis (DEP) is defined as the motion of an electrically polarisable particle in a non-uniform electric field [4]. DEP is commonly used in lab on chips devices to perform cell sorting and characterization. The dielectrophoretic force applied to the cells depends on their intrinsic electric and geometrical properties [5,6]. Consequently, cells of different properties can thus be separated using this principle.

DEP actuation can be used to perform different types of cell manipulations. Cells can be trapped between electrodes and immobilized [7]. Cells can be moved using traveling wave DEP [8]. Heterogeneous cell samples can be sorted by DEP field-flow fractionation [9] or multi-frequency DEP [10]. The selectivity of cell sorting based on DEP actuation highly depends on the difference of geometrical and electric properties of the cells. Cells with similar electric properties, such as cancer cells originated from different tissues [11], cannot be sorted using this technique. The fluorescent activated cell sorters propose an other way, which decouple the detection principle from the separation principle [12]. In these devices, each cell is analyzed based on fluorescence of dyes used to label the cells (detection stage) and then each cell trajectory is controlled actively to orient it in the selected channel (sorting stage). This sorting stage is often performed using dielectrophoretic actuation. For example, Tian et al. [13], Holmes et al. [14] and Ahn et al. [15] propose dielectrophoretic systems with two pairs of electrodes switched on or off depending on the cell’s destination.

We propose to improve DEP sorting systems to sort several cells at the same time. This requires to be able to precisely plan and control the position of several cells in a channel simultaneously. We propose to use closed-loop control in order to define the voltage applied to each electrode in real-time based on the information of the current position of the cells given by exteroceptive sensors, such as cameras. In literature, few articles have demonstrated experimental closed-loop control on DEP actuation [16,17,18]. Recently, Zemánek et al. [19] proposes a simple geometry, based on a parallel electrode array, similar to those of traveling wave systems. Contrary to traveling waves systems, they precisely control the voltage on each electrode in function of the position of the cells. They demonstrate the independent manipulation of three particles. The positions of the particles are monitored in real-time using a camera. This achievement illustrates the interest of using closed-loop control (i.e., of adjusting in real time the force applied to the cells based on a real-time measurement of the position of the cells). However, closed-loop systems need a mathematical model to compute the adequate input (namely the voltage applied to each electrode) for a given output (namely the position of the cells). Previously cited works, [16,17,18,19], used numerical model, leading to computing times of tens of milliseconds.

To reduce the computing time, we propose to use an analytical model of the dielectrophoretic force requiring an analytical model of the electric field generated by the electrodes. Several approaches reported in literature deal with providing analytical models of the electric field for traveling wave dielectrophoresis. The Green’s theorem is one way to calculate the electric field using appropriate Green’s functions [20]. Fourier series could be also a convenient tool to convert a periodic boundary condition, namely the voltage applied on the electrodes, into a sum of exponential functions enabling to solve a partial differential equation system, namely the electric field in the system [21,22]. Schwarz-Christoffel mapping is a conformal transformation used to rearrange the boundary conditions in order to simplify the resolution of the electric field in the system [23]. However, these models based on a period representation are suited only for traveling wave systems, and are not valid if the magnitude of the electric potential vary on each electrode. For more complex systems, the Laplace’s equation defining the electric field as a linear system of the voltage on each electrodes can be exploited. Classical resolution methods consist of adding the contribution of each electrode. In this purpose, a generic formulation of the electric field produced by two opposite electrodes (one electrode is placed on the bottom of the channel, and the second one at the top) has been proposed by Nerguizian [24]. However, it assumes a symmetric distribution of the electric field on each side of the electrodes, which is not respected near the channel walls. Gurtner et al. [25] proposed a semi-analytical model of the electric potential, based on Green’s functions. However, its accuracy is also limited near the channel walls. For this reason, Song proposes an analytical model, based on Fourier series, which uses an artificial neural network to learn the coefficients of the series [26]. Its implementation based on learning is unfortunately complex.

The aim of this paper is to propose a model dedicated to closed-loop control enabling to compute the electric field and dielectrophoretic force applied to cells. Closed-loop control consists in adjusting in real-time the voltage applied to the electrodes based on a feedback (usually the position of the cells obtained from images given by a camera). It ensures that the cells can follow reference trajectories despite perturbations. However, it requires to compute in real time a model of the forces applied to the cells. To ensure fast computing and resources-saving, we propose an original model, based on Fourier series, formulated as a product of matrices to separate the terms related to the geometry of the system (that can be precomputed), the input variables (voltage applied to each electrode) and the cell positions. The electric field can thus be evaluated at the given position of the cells, without the need of computing the electric potential in the whole workspace.

## 2. Electric Field Produced by an Electrode Array

### 2.1. Cell Sorting Using Parallel Electrode Arrays

**Figure 1:**
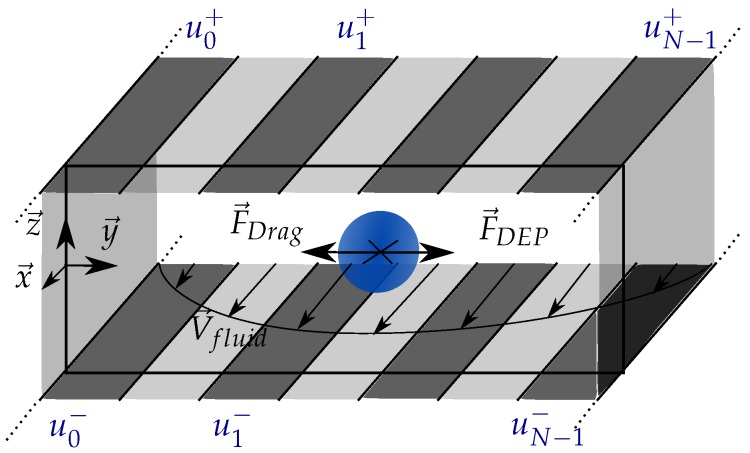
Sketch of the proposed sorting stage, based on electrode arrays, integrated in a fluidic chip. The cells flow through a channel due to the fluid motion. The lateral position of the cells is controlled by the electric potential applied on each electrode at the top and the bottom of the channel. Moving the cell along the *y* axis enables to collect them in different reservoirs to sort them.

The sorting stage proposed in this paper is presented in Figure 1. The cells flow through a fluidic channel having an infinite length following the $\vec{x}$ direction, a width *L* following the $\vec{y}$ direction and a height *h* following the $\vec{z}$ direction. Long electrodes are deposited along the channel, at the top and the bottom. As the system is invariant following the $\vec{x}$ direction, it can be reduced to a 2D problem in the $\left(0,\vec{y},\vec{z}\right)$ plane.

To sort the cells, the sorting stage must control their lateral position *y* in the channel, so that they can be collected in dedicated reservoirs (not represented in Figure 1). Since their position must be precisely controlled, closed-loop control based on exteroceptive sensors (such as a camera) will be used in future works to monitor the position of the cells in the channel in real-time. To perform this closed-loop control, it is necessary to get a model of the force applied to the cells. In the system, the cells experience gravity, buoyancy (both well known), fluid drag force and dielectrophoretic force. The drag force is given by Stokes’s law [27]. The dielectrophoretic force is computed from the electric potential’s spatial derivatives, by the classic formulation expressed by Pohl [28]. However, it requires knowing the electric field produced by the electrode arrays.

The goal of this paper is thus to propose an analytical model of the dielectrophoretic force field produced by two planes of respectively $N^{+}$ and $N^{-}$ parallel electrodes on which arbitrary voltages can be set (contrary to what is done in traveling wave). The model must be fast to compute in order to be used in real-time to control the particle position in closed-loop.

The model of the electric potential produced by one electrode array (bottom array) is given in Section 3. This result is generalized in Section 4 to the potential produced by two arrays (situated at the bottom and the top of the channel), and the dielectrophoretic force applied to the cells is given. Section 5 discusses the performances of the proposed model for real-time closed-loop control.

### 2.2. Generic Solution of the Electric Potential Produced by an Electrode Array Expressed as a Fourier Series

To get an efficient model for closed-loop control, it is necessary to separate the terms depending on the geometry of the system (that can be precomputed), the terms depending on the position of the cell (which changes at each time step) and the terms depending on the input variables (namely the voltage applied to each electrode). This section proposes a formulation of the electric potential as a product of matrices that decouples the above-mentioned terms based on Fourier series.

The electric potential generated by an array of parallel electrodes has to satisfy the Maxwell’s equations. For currents and frequencies typically found in dielectrophoretic systems, the electric potential can be reduced to the quasi-static form of the Maxwell’s equations. This means that spatial and temporal descriptions of the electric field are decoupled. For any excitation u(t), the electric potential can be expressed as ϕ(y,z,t)=α(y,z)u(t). As stated by Green [29], for a linear homogeneous medium in a quasi-static mode, the electric potential satisfies the Laplace’s equation:

(1) $$\nabla^{2}\phi =0,$$

where $\nabla^{2}$ denotes the laplacian operator. As this differential equation is linear, the electric potential generated by all the electrodes can be written as a linear combination of the potential generated by each electrode. In the following the electric voltage applied to the n^th^ electrode is named $u_{n}\left(t\right)$. The electric potential can be expressed as:

(2) $$\phi \left(y,z,t\right)=\sum_{n}\alpha_{n}\left(y,z\right)u_{n}\left(t\right),$$

where $\alpha_{n}\left(y,z\right)$ is the elementary voltage generated by the electrode *n* at the point (y,z) (in volt per volt). Classical numerical identification methods solve directly $\alpha_{n}\left(y,z\right)$ applying 1 V to the n^th^ electrode and zero to the others [16]. In this paper, we propose a different approach to decouple the terms which depend on the system geometry, the control parameter and the current position of the controlled particle. We make the following assumptions in order to build an analytical expression of the elementary voltage $\alpha_{n}\left(y,z\right)$:

1. the spatial variables can be separated:
  (3) $$\alpha_{n}\left(y,z\right)=Y_{n}\left(y\right)\;\cdot \;Z_{n}\left(z\right);$$
2. the dependence along the $\vec{y}$ axis can be expressed as a Fourier series:
  (4) $$Y_{n}\left(y\right)=\sum_{p}a_{p,n}\;e^{i\omega_{p}y},$$

with $a_{p,n}$, the Fourier coefficient of the p^th^ angular frequency $\omega_{p}$. The first assumption is confirmed by the existence of a solution, Equation (6). The convergence of the series toward $Y_{n}\left(y\right)$ will be verified in Figure 4. Combining Equations (1) and (2) imply that the laplacian of each elementary potential, $\nabla^{2}\alpha_{n}\left(y,z\right)$ is null. Consequently, based on Equations (3) and (4):

(5) $$\forall n,\;\nabla^{2}\alpha_{n}\left(y,z\right)=0=\sum_{p}a_{p,n}\;e^{i\omega_{p}y}\left(-\omega_{p}^{2}Z_{n}\left(z\right)+\frac{d^{2}Z_{n}\left(z\right)}{dz^{2}}\right).$$

Equation (5) has an infinite number of solutions $Z_{n}\left(z\right)$. We choose the solution giving $Z_{n}\left(z=h\right)=0$ and $Z_{n}\left(z=0\right)=1$. This solution has been selected since it satisfies the boundary condition (11) and simplifies Equation (12). Thus, the electric potential in the system is:

(6) $$\phi \left(y,z,t\right)=\sum_{n}\left(a_{0,n}\frac{h-z}{h}+\sum_{p\neq 0}a_{p,n}e^{i\omega_{p}y}\;\frac{e^{\omega_{p}\left(2h-z\right)}-e^{\omega_{p}z}}{e^{2\omega_{p}h}-1}\right)u_{n}\left(t\right).$$

This double sum formulation can also be expressed as a matrix product:

(7) $$\phi \left(y,z,U_{\left[N\right]}\left(t\right)\right)=\;{{}^{t}e}_{\left[P\right]}\left(y,z\right)\;\cdot \;A_{\left[P\times N\right]}\;\cdot \;U_{\left[N\right]}\left(t\right),$$

where $U_{\left[N\right]}\left(t\right)$ is a (*N*,1) vector composed of the electric potential, $u_{n}\left(t\right)$, applied to each electrode, $A_{\left[P\times N\right]}$ is a (*P*, *N*) matrix composed of all the Fourier coefficients $a_{p,n}$ and $\;{{}^{t}e}_{\left[P\right]}\left(y,z\right)$ is the transpose vector of the *P* exponential terms evaluated at the point of application (y,z). This formulation is interesting for automation application because the control parameters of the system, $U_{\left[N\right]}\left(t\right)$, the geometry of the system, $A_{\left[P\times N\right]}$, and the position of the particle (y,z) are decoupled. Furthermore, contrary to numerical methods, which need to solve the electric field in the whole space, the vector function $\;{{}^{t}e}_{\left[P\right]}\left(y,z\right)$ enables to compute easily the electric potential and all its spatial derivatives only in the point of interest (y,z). The gain in computing speed will be evaluated at the end of this article.

## 3. Analytical Formulation of the Electric Potential Produced by One Plane of Electrode Array

**Figure 2:**
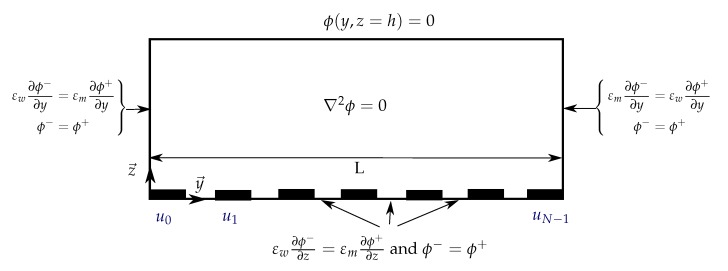
Schematic representation of the boundary conditions determining the electric potential above an array of electrodes. $\phi^{+}$ and $\phi^{-}$ are the electric potentials on each side of the boundary. $\varepsilon_{m}$ is the permittivity of the fluid in the channel and $\varepsilon_{w}$ is the permittivity of the wall.

The goal of this section is to get an analytical expression of the Fourier coefficients $a_{p,n}$ of the matrix $A_{\left[P\times N\right]}$ of the electric potential produced by a single electrode array (Figure 2).

### 3.1. Boundary Conditions of the Electric Potential

The previous assumption, Equation (4), and the resulting formulation of the electric potential (7) are valid only if there exist Fourier coefficients, $a_{p,n}$, satisfying the boundary conditions of the system (Figure 2).

The exact boundary conditions are assumed to be as follows. The array of electrodes at the bottom is considered as a perfect conductor. The electric potential on the electrodes is uniform and well known. The influence of the double layer on the electrode surface is neglected. The electric potential is grounded at a height, h, above the electrode array. In the next section, this will be used to superimpose the solutions issued from each electrode planes to compute the force produced on a cell by two parallel plans of electrodes. Finally, all other walls are considered as perfect insulators and without charge accumulation. Thus, the electric displacement across these boundaries is uniform, and the potential is continuous.

**Figure 3:**
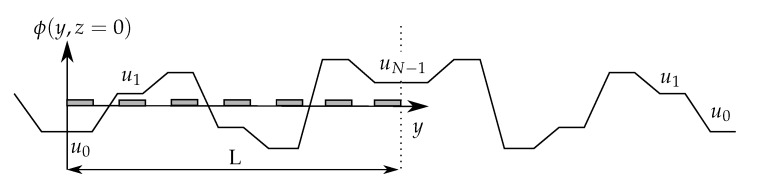
Transcription of the boundary conditions to the potential approximate on the electrode plane. The non-flux condition on the lateral wall is seen as a symmetry. The potential between each pair of electrode is approximated by a linear function.

The symmetry used in [20,23] to solve the problem using Green’s theorem or Schwarz-Christoffel method are no more valid. In order to find an analytical solution of the problem using Fourier series, the Neumann type boundary conditions are converted in conditions on the electric potential. Between the electrodes, the electric potential is assumed to be linear, as in [21,29]. On lateral walls the condition of uniform electric displacement induces a reflection of the electric field. This can be solved using the method of image charges [30]. The coefficient of reflection, $\frac{\varepsilon_{m}-\varepsilon_{w}}{\varepsilon_{m}+\varepsilon_{w}}=0.9$, where $\varepsilon_{m}=78$ is the relative medium permittivity and $\varepsilon_{w}=4$ is the relative permittivity of the wall made of SU-8, approximates 1. Thus, the boundary condition on the lateral walls can be seen as a symmetry condition. The electric potential in the system is virtually expanded to the whole space by repetitive symmetry along the lateral borders forming a periodic pattern (see Figure 3).

These assumptions will be discussed at the end of this paper by comparing the analytical model obtained using these assumptions to a numerical model implemented in COMSOL software, using the previously cited exact boundary conditions.

Assuming that there are N electrodes regularly spaced in the channel of width L, as in classical setup [20,21], the electrode width and the space between each electrode is $\lambda =\frac{L}{2N-1}$. The boundary conditions can be analytically expressed as follows:

1. the electric potential on the n^th^ electrode is $u_{n}$, ∀0≤n≤N−1, y∈[2nλ;(2n+1)λ]
  (8) $$\phi \left(y,z=0\right)=u_{n};$$
2. the electric potential between two electrodes is linear, ∀0≤n≤N−2, y∈[(2n+1)λ;(2n+2)λ]
  (9) $$\phi \left(y,z=0\right)=u_{n}+\frac{u_{n+1}-u_{n}}{\lambda }\left(y-\left(2n+1\right)\lambda \right);$$
3. the electric potential is symmetrical across lateral boundaries, y=0, y=L, …, y=kL,
  (10) ϕ(kL+y,z)=ϕ(kL−y,z),k∈Z;
4. the potential is grounded at height h,
  (11) ϕ(y,z=h)=0.

The last condition is satisfied as we already choose the adequate solution to Equation (5). Equations (8)–(10) define by parts the potential on the lower boundary as a periodic function. The Fourier series will enable to transform this set of equations into a single function defined for all *y*.

### 3.2. Analytical Formulation of the Fourier Coefficients Satisfying the Boundary Conditions

Equation (7) gives the general formulation of the electric potential generated by one plane of array of electrodes ϕ(y,z,t) . Equations (8)–(10) give the boundary conditions that the electric potential must satisfy. This section aims at determining the coefficients $a_{p,n}$ of the matrix $A_{\left[P\times N\right]}$ defined in Equation (7) obeying the boundary conditions.

According to Equation (6) and the choice of $Z_{n}\left(z=0\right)=1$, the electric potential on the lower boundary is:

(12) $$\phi \left(y,z=0\right)=\sum_{n}\sum_{p}u_{n}\left(t\right)\;a_{p,n}\;e^{i\omega_{p}y}=\sum_{p}\overset{\mathrm{Fourier}\mathrm{coefficients}}{\overset{︷}{\sum_{n}u_{n}\left(t\right)\;a_{p,n}}}\;e^{i\omega_{p}y}.$$

According to Equation (10), the electric potential on the lower boundary is a periodic function of period 2L. We consequently choose:

(13) $$\omega_{p}=2p\pi /2L.$$

Moreover, Equation (12) describes a Fourier series in which the Fourier coefficients are $\sum_{n}u_{n}\left(t\right)\;a_{p,n}$. By definition, the p^th^ Fourier coefficient is given by:

(14) $$\sum_{n}u_{n}\left(t\right)\;a_{p,n}=\frac{1}{2L}\int_{-L}^{L}\phi \left(y,z=0\right)e^{-i\omega_{p}y}dy.$$

The function ϕ(y,z=0) is defined by parts (see Equations (8) and (9)). The integral (Equation (14)) can be expressed as the sum of the integral of each part:

(15) $$\begin{array}{cc} \sum_{n}u_{n}\left(t\right)a_{p,n}= & \frac{1}{2L}\left[\sum_{n=0}^{N-1}\left[\int_{2n\lambda }^{(2n+1)\lambda }u_{n}e^{-i\omega_{p}l}dl+\int_{-(2n+1)\lambda }^{-2n\lambda }u_{n}e^{-i\omega_{p}l}dl\right]\right.\\ & \;+\sum_{n=0}^{N-2}\left[\int_{(2n+1)\lambda }^{(2n+2)\lambda }\left(u_{n}+\frac{u_{n+1}-u_{n}}{\lambda }\left(l-\left(2n+1\right)\lambda \right)\right)e^{-i\omega_{p}l}dl\right.\\ & \;+\left.\left.\int_{-(2n+2)\lambda }^{-(2n+1)\lambda }\left(u_{n+1}+\frac{u_{n}-u_{n+1}}{\lambda }\left(l+\left(2n+2\right)\lambda \right)\right)e^{-i\omega_{p}l}dl\right]\right] \end{array}.$$

The integration of the first set of integral relative to Equation (8) is obvious. The second set relative to the linear approximation can be solved using $\int_{a}^{b}l\;\cdot \;e^{-i\omega l}dl=\frac{1}{\omega^{2}}{\left[\left(i\omega l+1\right)\;\cdot \;e^{-i\omega l}\right]}_{a}^{b}$. Factorizing $u_{n}\left(t\right)$, the analytical formulation of the Fourier coefficients $a_{p,n}$ is given in Table 1, with $\gamma_{p}=\frac{p\pi }{2N-1}$.

The electric potential ϕ(y,z,t) in the whole space produced by the bottom electrode array is given by Equation (7) in which the parameters $\omega_{p}$ and $a_{p,n}$ are defined in Equation (13) and Table 1.

To verify the convergence of the Fourier series, the analytical expression given above is implemented numerically for different values of the length, *P*, of the Fourier series:

(16) $$\phi \left(y,z=0,t\right)=\sum_{p=-P}^{P}\sum_{n}u_{n}\left(t\right)a_{p,n}e^{i\omega_{p}y}.$$

**Figure 4:**
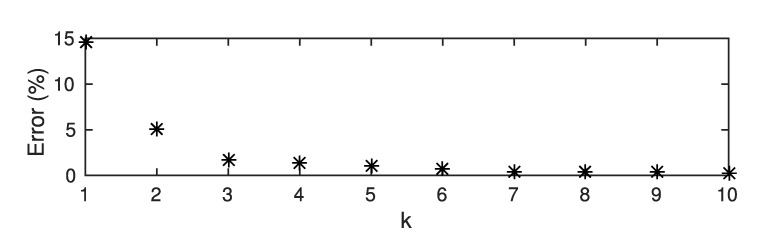
Convergence of the Fourier series, $\sum_{n}\sum_{p}u_{n}\left(t\right)\;a_{p,n}\;e^{i\omega_{p}y}$, toward the approximate boundary condition, ϕ(y,z=0), defined by Equations (8)–(10). The dots correspond to the relative root mean square error in percent for different values of P=k∗N.

Results are given in Figure 4 for an arbitrary input command $U_{\left[N\right]}$. As *P* increases the Fourier series converges to the boundary condition. Equation (4) is thus valid, as well as the resulting formulation, Equation (7).

It can be noticed that the Fourier coefficients, $a_{p,n}$, expressed by the set of equations summarized in Table 1, depends only of *N*, the number of electrodes, which is fixed for a given chip. Their calculations do not need a learning process as in [26]. To increase the speed of the feedback loop in real-time applications, these coefficients can be pre-computed and stored in memory.

## 4. Generalization to the Analytical Formulation of the Electric Potential and the Dielectrophoretic Force Produced by Two Planes of Electrode Arrays

The analytical formulation of the electric potential produced by one plane of electrode array has been presented in the previous section. This section aims at generalizing this result to the electric potential produced by two planes of electrode arrays, which is the geometry proposed in Section 2.1. The expression of the electric potential is then used to express the dielectrophoretic force applied to the cells.

### 4.1. Generalization of the Electric Potential to the Case of Two Parallel Planar Arrays of Electrodes

It is interesting, from the perspective of cells sorting, to get a second plane of electrodes as depicted in Figure 1. Adding this second plane enables to control the altitude *z* of the cells. This system can also be seen as a particular form of multipolar system for which the simultaneous control of a multiple particles has been proved [18].

The electrode arrays are supposed to be placed on each face of the channel at z=±h/2. The number of electrodes on the upper (resp. lower) plane is $N^{+}$ (resp. $N^{-}$). $N^{+}$ can be different from $N^{-}$. This system is similar to the previous one. The only difference concerns the boundary condition (11) which sets a grounded potential at a height h above the electrode array. It is replaced by a set of conditions similar to (8)–(10) expressing the electric potential on the second electrode array. Thus the electric potential induced by this configuration is the superposition of the electric potential induced by each electrode arrays and can be written as:

(17) $$\begin{array}{c} \begin{array}{cc} \phi (y,z,U_{\left[N^{+}+N^{-}\right]}) & =\left[{{}^{t}e}_{\left[P\right]}\left(y,\frac{h}{2}-z\right)\cdot \;A_{\left[P\times N^{+}\right]}^{+}\;\cdot \;U_{\left[N^{+}\right]}^{+}\right]+\left[\;{{}^{t}e}_{\left[P\right]}\left(y,\frac{h}{2}+z\right)\;\cdot \;A_{\left[P\times N^{-}\right]}^{-}\;\cdot \;U_{\left[N^{-}\right]}^{-}\right]\\ & =\left(\begin{array}{cc} \;{{}^{t}e}_{\left[P\right]}\left(y,\frac{h}{2}-z\right) & \;{{}^{t}e}_{\left[P\right]}\left(y,\frac{h}{2}+z\right) \end{array}\right)\;\cdot \;\left(\begin{array}{cc} A_{\left[P\times N^{+}\right]}^{+} & 0\\ 0 & A_{\left[P\times N^{-}\right]}^{-} \end{array}\right)\;\cdot \;\left(\begin{array}{c} U_{\left[N^{+}\right]}^{+}\\ U_{\left[N^{-}\right]}^{-} \end{array}\right)\\ & =\;{{}^{t}e}_{\left[2P\right]}\left(y,z\right)\;\cdot \;A_{\left[2P\times \left(N^{+}+N^{-}\right)\right]}\;\cdot \;U_{\left[N^{+}+N^{-}\right]} \end{array} \end{array}$$

where $A_{\left[P\times N^{+}\right]}^{+}$ (resp. $A_{\left[P\times N^{-}\right]}^{-}$) is the matrix corresponding to the terms describing the geometry, given in Table 1, of the upper (resp. lower) array, and $U_{\left[N^{+}\right]}^{+}$ (resp. $U_{\left[N^{-}\right]}^{-}$) is the matrix corresponding to the electric potential on the upper (resp. lower) electrode array. Equation (17) is the generalization of Equation (7). It provides a generic expression of the electric potential produced by two planar electrode arrays.

### 4.2. Dielectrophoretic Force Formulation

Knowing the electric potential generated by the system (17), the related dielectrophoretic force can be expressed using the classical formulation introduced by Pohl [28]:

(18) FDEP=2πϵ0ϵmr3Re(β)∇(∇ϕ)2,

where $\epsilon_{0}$ is the vacuum permittivity, $\epsilon_{m}$ is the relative medium permittivity, *r* is the particle radius and Re(β) is the real part of the Clausius-Mossotti polarization factor defined as

(19) $$\beta =\frac{\epsilon_{p}^{*}-\epsilon_{m}^{*}}{\epsilon_{p}^{*}+2\epsilon_{m}^{*}},$$

where $\epsilon_{p}^{*}=\epsilon_{p}-i\sigma_{p}/\upsilon$ is the complex permittivity of the particle, $\sigma_{p}$ is its conductivity and υ is the angular frequency of the applied electric field. $\epsilon_{m}^{*}$, the complex permittivity of the medium, is defined similarly. The vector function $\nabla {\left(\nabla \phi \right)}^{2}$ can be computed based on the expression of the electric potential determined in the previous sections as follows. On the $\left(0,\vec{y},\vec{z}\right)$ plane:

(20) $$\nabla {\left(\nabla \phi \right)}^{2}=2\left(\begin{array}{c} \frac{\partial^{2}\phi }{\partial y^{2}}\;\cdot \;\frac{\partial \phi }{\partial y}+\frac{\partial^{2}\phi }{\partial y\partial z}\;\cdot \;\frac{\partial \phi }{\partial z}\\ \frac{\partial^{2}\phi }{\partial z\partial y}\;\cdot \;\frac{\partial \phi }{\partial y}+\frac{\partial^{2}\phi }{\partial z^{2}}\;\cdot \;\frac{\partial \phi }{\partial z} \end{array}\right).$$

Using Equation (17), the derivatives of the electric potential are:

(21) $$\frac{\partial \phi }{\partial .}=\frac{{\partial \;{}^{t}e}_{\left[2P\right]}}{\partial .}\left(y,z\right)\;\cdot \;A_{\left[2P\times \left(N^{+}+N^{-}\right)\right]}\;\cdot \;U_{\left[N^{+}+N^{-}\right]}$$

According to Equations (6) and (17),

(22) $$\begin{array}{cc} {{}^{t}e}_{\left[2P\right]}\left(y,z\right)= & \left(e^{i\omega_{P}y}\left(\frac{e^{\omega_{P}\left(3h/2+z\right)}-e^{\omega_{P}\left(h/2-z\right)}}{e^{2\omega_{P}h}-1}\right)\cdots \frac{1}{2}+\frac{z}{h}\cdots e^{i\omega_{-P}y}\left(\frac{e^{\omega_{-P}\left(3h/2+z\right)}-e^{\omega_{-P}\left(h/2-z\right)}}{e^{2\omega_{-P}h}-1}\right)\right.\\ & \left.e^{i\omega_{P}y}\left(\frac{e^{\omega_{P}\left(3h/2-z\right)}-e^{\omega_{P}\left(h/2+z\right)}}{e^{2\omega_{P}h}-1}\right)\cdots \frac{1}{2}-\frac{z}{h}\cdots e^{i\omega_{-P}y}\left(\frac{e^{\omega_{-P}\left(3h/2-z\right)}-e^{\omega_{-P}\left(h/2+z\right)}}{e^{2\omega_{-P}h}-1}\right)\right). \end{array}$$

The computation of its derivatives $\frac{{\partial \;{}^{t}e}_{\left[2P\right]}}{\partial .}\left(y,z\right)$ is straightforward since it is a combination of derivative of exponential functions.

Thus, the system is fully described by an analytical expression depending on the actuation command, $U_{n}\left(t\right)$. In future works, this model will be inverted to perform closed loop control. This control law will be implemented in the experimental system described in Section 2.1 to sort cells based on external analysis, such as the results of a fluorescence analysis.

## 5. Results and Discussion

### 5.1. Comparison between the Analytical Model and a Numerical Simulation

The analytical formulation proposed in this paper is compared to a numerical simulation performed with COMSOL multiphysics software. It is a common practice to compare the value of the dielectrophoretic force obtained by analytical models with the ones obtained using a numerical software. To name only a few, [22] compares the analytical expression of the force to a numerical simulation, [23,24] compare the analytical solution to a finite element simulation performed using COMSOL software and [26] compares the analytical expression of the force to a numerical simulation performed with CFD-ACE software. In addition, a good agreement between the numerical simulation and the experimental results is shown in [24]. We thus made the choice, in this article, to take as a reference the numerical simulation.

**Figure 5:**
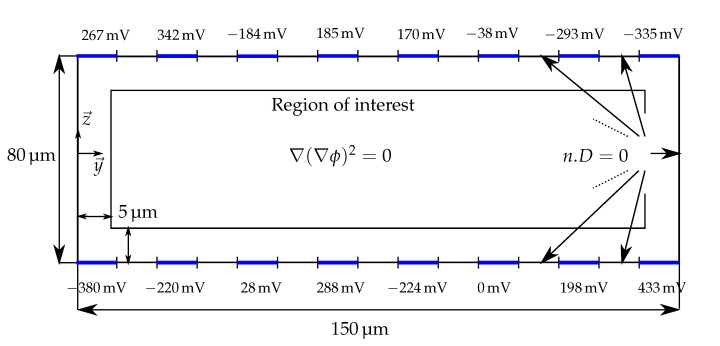
Schematic representation of the parameters used for the simulation with COMSOL software. D=ϵE represents the electric field displacement and *n* the normal to the boundary.

**Figure 6:**
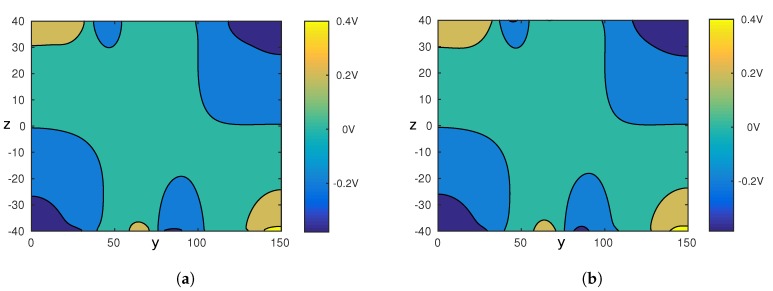

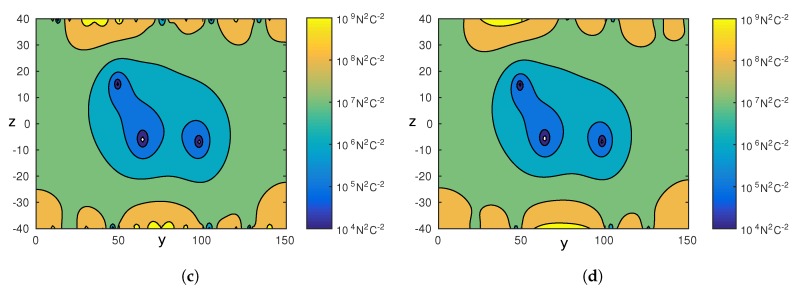
(**a**,**c**) are issued from COMSOL model. (**b**,**d**) are issued from the analytical model proposed in this paper. Figures at the top row show the electric potential for a random voltage applied to each electrode. Figures on the bottom show the corresponding squared electric field amplitude ${\left(\nabla \phi \right)}^{2}$. The parameters of the chip are the following: width L=150µm, height h=80µm, number of upper and lower electrodes $N^{+}=N^{-}=8$. The Fourier series is computed for P=2N. (**a**) Electric potential, FEM; (**b**) Electric potential, analytical model; (**c**) Squared electric field, FEM; (**d**) Squared electric field, analytical model.

The model simulated is depicted in Figure 5. The system is composed of eight electrodes of 10µm width, uniformly spaced by 10µm on the top and bottom of the fluidic channel. For interested readers, Green [29] studied the influence of the ratio between the length of the electrodes and the gap between two electrodes. The channel is 150µm width and 80µm height. The dielectrophoretic force is computed considering a particle of 8µm diameter, in a medium with a permittivity $\epsilon_{m}=78$. The Clausius-Mossotti factor, which depends on the electric field frequency and the medium conductivity, is set to −0.5. The electric potential is computed as a solution of the Laplace Equation (1). By default the boundary conditions consider that there is no charge accumulation. The potential applied to the electrodes is given in Figure 5. The Fourier series is computed for P=2N ($N=N^{+}=N^{-}=8$). Figure 6 presents the results. The left column of Figure 6 corresponds to the numerical simulation, and the right part is the analytical model given in Equation (17). These figures show similar pattern in form and amplitude for both the electric potential and the squared electric field generated by two arrays of electrodes.

**Figure 7:**
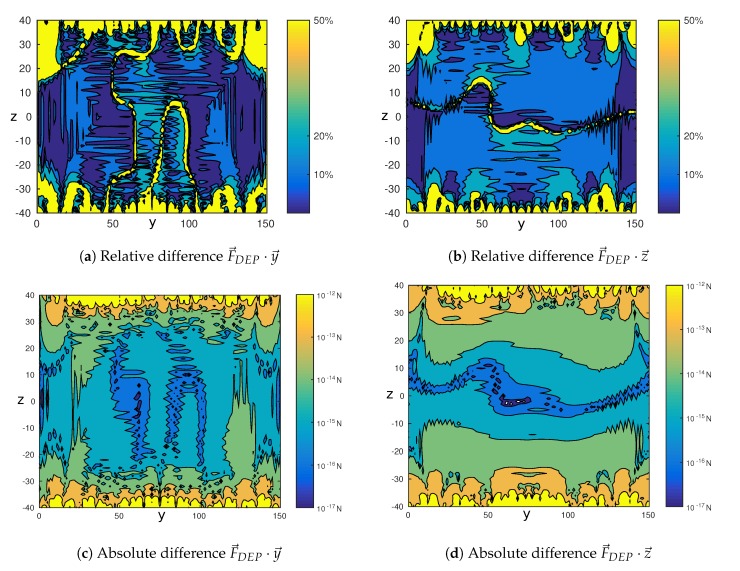

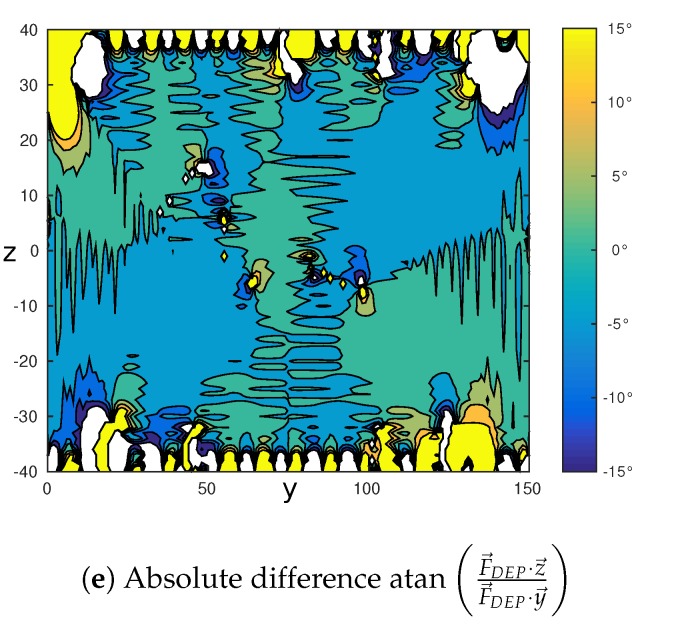
Difference between the dielectrophoretic force computed with COMSOL software according to the parameters given in Figure 5 and the one computed with Matlab according to the analytical model proposed in this paper. (**a**,**b**) give the relative difference in percentage and (**c**,**d**) give the absolute difference on the dielectrophoretic force in Newton along both the $\vec{y}$ and $\vec{z}$ axes. (**e**) gives the error on the force orientation in degrees.

Figure 7 shows the relative (top) and absolute (bottom) error on the dielectrophoretic force, along the $\vec{y}$ axis on the left and the $\vec{z}$ axis on the right. These figures show a relative error of less than 20% in magnitude and an absolute error of less than 5 degrees in orientation in most of the space. The relative error is locally high (200%) where the force is close to zero. This is due to the fact that the absolute error is divided by a force almost null, leading to a large relative error, despite a moderated absolute error. The error is also important near the electrodes. This is due to the approximation made on the boundary conditions (Figure 3). This is not a critical point as in applications using negative dielectrophoresis, the particles flow away from the electrodes. In addition, the classical formulation of dielectrophoresis is no more valid close to the walls [31]. In the following, the study is reduced to a region of interest situated in the center of the channel defined in Figure 5.

### 5.2. Influence of the Length of the Fourier Series

To be used in real-time applications, the Fourier series used to compute the electric potential has to be truncated to the minimum number of terms. The critical point to precisely control in closed-loop the position of the cells in the chip is the prediction of the direction of dielectrophoretic force $F_{DEP}$. The influence of the length of the Fourier series on the prediction of the direction of the force is studied in Table 2. This table presents the root mean square difference between the arctangent of the ratio $F_{DEP}\;\cdot \;\vec{z}$/$F_{DEP}\;\cdot \;\vec{y}$ computed from the COMSOL model and from the analytical model proposed in this paper. The two models are compared for different values of the parameters $N=N^{+}=N^{-}$, the number of electrodes and *P*, the number of terms of the Fourier series for a channel width of 150µm and height 80µm. This analysis is made in the region of interest defined in Figure 5. The results are based on 800 simulations for different combinations (P,N), for which different electric potentials are applied on the electrodes. An example is given in Figure 7e. It is obtained using the simulation parameters given in Section 5.1.

According to Table 2, the convergence of the analytical model toward the numerical model improves until the length of the Fourier series reaches P=2N. The average error in Table 2, 30°, is higher than the error in most of the region of interest, as shown in Figure 7e. This is due to the fact that the angle depends on the ratio between ${\vec{F}}_{DEP}\;\cdot \;\vec{z}$ and ${\vec{F}}_{DEP}\;\cdot \;\vec{y}$. When the magnitude of ${\vec{F}}_{DEP}\;\cdot \;\vec{y}$ is small isolated errors up to 180 degrees arise, leading to high average errors.

### 5.3. Computing Time of the Fourier Series

Beside the precision of the force estimation, a second critical point for real-time control is the computing time. The size of $A_{\left[P\times N\right]}$ being (2P+1)×N, this matrix does not need a lot of space in memory and can be pre-computed to improve the calculation in real-time application. Table 3 summarizes the average time to compute the dielectrophoretic force applied to one point (*y*,*z*) by our method using Matlab. The mean value is 0.215 ms. This is 5000 times faster than the computing time using COMSOL FEM solver (>1 s).

### 5.4. Discussion

As mentioned in Section 3.1, the analytical formulation proposed in this paper is based on approximated boundary conditions, according to Figure 3. However the COMSOL numerical simulations are performed with the exact boundary conditions according to Figure 5. The good agreement between those two models, highlighted by both the qualitative and quantitative comparison validates the assumptions made in Section 3.1 on the boundary conditions.

The error on the calculation of the dielectrophoretic force magnitude is generally under 20% in the region of interest. This correlation is in accordance with Green’s work, [29], which compares Fourier series and FEM for traveling wave with linear approximation of the boundary condition. These two works, Green’s and ours are less accurate than the approach proposed by Song et al. [26]. Song improves the accuracy near the electrode using an artificial neural network to break down the boundary conditions, allowing to compute higher orders of the Fourier series. However, for automation application, 20% of error in the field magnitude can easily be corrected using closed-loop and the priority is given to the simplicity of the model.

The analytical model proposed in this paper, to compute the electric potential and then the dielectrophoretic force generated by an electrode array, gives a good enough estimation of the force magnitude and orientation. In addition, this model is fast to compute and does not need a lot of space in memory. These skills make this model a perfect candidate for automation system. Its analytical formulation enables to use it for any setup based on 1D electrode arrays, similar to traveling wave systems. This method can be extended to 2D electrode arrays, similar to [32], using multivariate Fourier series.

**Figure 8:**
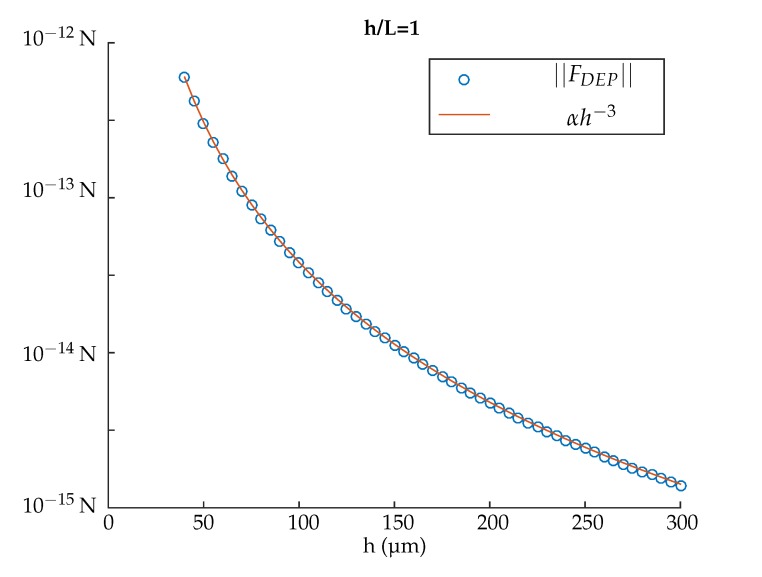
Example of the analysis of the impact of one geometrical parameters on the DEP force: Influence of the height *h* of the channel, for h/L=1. The plotted force values correspond to the mean value of the quadratic norm of the dielectrophoretic force on the segment (y∈[L/3;2L/3],z=0). The number of electrodes is $N^{+}=N^{-}=8$ and the voltage applied on them is detailed in Figure 5.

The proposed model can also be used during the design process in order to test the impact of parameters on DEP force distribution. The Figure 8 shows an example: the influence of the height *h* of the channel on the magnitude of the dielectrophoretic force considering a ratio h/L=1. The plotted values correspond to the mean value of the quadratic norm of the dielectrophoretic force on the segment (y∈[L/3;2L/3],z=0). Numbers of electrodes are $N^{+}=N^{-}=8$, and the voltage applied on them are detailed in Figure 5. In this example, the magnitude of the dielectrophoretic force is proportional to $\left|\right|F_{DEP}\left|\right|=K/h^{3}$, when h=L. The factor *K* is a function of the number of electrodes, the voltage applied on each electrode, the position of the particle and the electric properties of the system.

Future works include the inversion of the model to perform closed loop control. This control law will be implemented and tested on an experimental device. It also requires to track in real time the positions of the cells. We plan to use a visual feedback provided by a camera to get these positions. The positions on the plane (i.e., along the *x* and *y* axes) can be obtained immediately by performing basic image processing. The z position is more challenging. Recently [33] proposed a solution based on a twin beam illumination or a holographic configuration of the optical system. However, since two arrays of electrodes are needed, it requires to get transparent electrodes to avoid occlusions of the cells. This can be obtained using transparent conducting oxides or transparent conducting ink based on carbon nanotubes.

## 6. Conclusions

Currently, most of the dielectrophoretic systems are actuated in open-loop. To guarantee the position of the cells in fluidic chips closed-loop control, which consists in adjusting the voltage applied to the electrodes in real-time in function of the actual position of the cells, is an attractive solution. However, it requires to get an analytical model of the force produced on the cells in function of the voltage applied to each electrode that can be inverted real-time.

In this paper, a methodology for computing the dielectrophoretic force produced by two parallel electrode arrays has been proposed and implemented. This methodology has the advantage to compute the electric potential as a product of three independent matrices which depend on the geometry of the chip, the potential applied on each electrode and the position of the point of interest. This model has two main advantages for automation applications. First, this matrix product is fast to compute. Second, the input of the system (voltage applied to the electrodes) is decoupled from the other terms. It will thus be of great interest for automation applications where the voltage to be applied to the electrodes to produce a desired dielectrophoretic force on the cells needs to be computed in real-time.

The methodology has been developed for one array of electrodes and then extended to two facing electrode arrays. Calculations for various electrode voltages show good agreement between this analytical model and classical FEM methods.

The next step of our work is to invert the model presented in this paper to get the voltage that should be applied to each electrode to apply a given force on a cell in the channel. A closed-loop model based control law will be implemented and tested on an experimental device to perform dielectrophoretic cell sorting. This work is thus the first step toward automated cell sorting using advanced control laws.

## Figures and Tables

**Table 1:** Fourier coefficients of the electric potential defined in Equation (7) and satisfying the boundary conditions (8)–(10).

$a_{p,n}$	p=0	p≠0
n=0	$\frac{3}{2}\times \frac{1}{2N-1}$	$\frac{2N-1}{p^{2}\pi^{2}}\left[\cos\left(\gamma_{p}\right)-\cos\left(2\gamma_{p}\right)\right]$
1≤n≤N−2	$\frac{2}{2N-1}$	$\begin{array}{cc} \frac{2N-1}{p^{2}\pi^{2}} & \left[-\cos\left(\left(2n-1\right)\gamma_{p}\right)+\cos\left(2n\gamma_{p}\right)\right.\\ & \left.+\cos\left(\left(2n+1\right)\gamma_{p}\right)-\cos\left(\left(2n+2\right)\gamma_{p}\right)\right] \end{array}$
n=N−1	$\frac{3}{2}\times \frac{1}{2N-1}$	${\left(-1\right)}^{p}\;\frac{2N-1}{p^{2}\pi^{2}}\left[\cos\left(\gamma_{p}\right)-\cos\left(2\gamma_{p}\right)\right]$

**Table 2:** Average difference between the dielectrophoretic force orientation predicted by our model and by a FEM model, based on 800 simulations for each combination (P,N). *N* is the number of electrodes and *P* is the length of the Fourier series. Each combination (P,N) is tested for different electric potential applied on the electrodes. The numerical model is meshed with free tetrahedral, size of the mesh <0.8µm.

θ	*N* = 5	*N* = 7	*N* = 9	*N* = 11	*N* = 13	*N* = 15
P=N	56.9°	44.8°	39.3°	35.0°	32.4°	31.1°
P=2N	42.2°	32.6°	28.7°	25.2°	24.0°	23.9°
P=3N	43.0°	32.3°	28.6°	25.4°	24.1°	23.8°
P=4N	41.5°	32.1°	28.2°	25.4°	24.2°	24.1°

**Table 3:** Average time to compute the dielectrophoretic force on one point (*y*,*z*), based on 800 simulations for each number of electrodes N. The precision of the Fourier series is P=2N.

	*N* = 5	*N* = 7	*N* = 9	*N* = 11	*N* = 13	*N* = 15
*t*	0.214 ms	0.208 ms	0.209 ms	0.217 ms	0.218 ms	0.221 ms

## Abbreviations

DEP	dielectrophoresis
FEM	finite element method
